# Endoscopic Mucosal Resection of Pancreatic Rest Presenting as a Sub-epithelial Nodule in the Gastric Antrum

**DOI:** 10.7759/cureus.50713

**Published:** 2023-12-18

**Authors:** Janak Bahirwani, Rodrigo Duarte-Chavez, Lisa Stoll, Ayaz Matin

**Affiliations:** 1 Gastroenterology, St. Luke's University Health Network, Bethlehem, USA; 2 Pathology, St. Luke's University Health Network, Bethlehem, USA

**Keywords:** endoscopic ultrasound, pancreatic neoplasia, endoscopic mucosal resection, gastric nodule, pancreatic rest

## Abstract

Pancreatic rest/ectopic pancreas is a rare condition. An 82-year-old male presented with abdominal pain and was found to have an antral nodule on esophagogastroduodenoscopy (EGD). An endoscopic ultrasound (EUS) was done and the nodule was resected. Histology showed ectopic pancreatic tissue with pancreatic intraepithelial neoplasia, PanIN-1 (low-grade dysplasia). This case highlights the importance of considering pancreatic rest as a differential in patients who present with a gastric sub-epithelial lesion and the associated finding of PanIN-1 highlights the importance of resecting such heterogeneous appearing lesions given the potential risk of progressing to pancreatic ductal adenocarcinoma (PDA).

## Introduction

Pancreatic rest, also known as heterotopic, ectopic, or aberrant pancreas, is the presence of ectopic pancreatic tissue outside the pancreatic parenchyma with a vascular and nerve supply separate from the normal pancreas. It can occur in all ages and has a prevalence of 0.55-13.7% in autopsies [1]. It is believed to arise during rotation of the foregut, when fragments of the pancreas become separated from the main body and are deposited at ectopic sites. It is predominantly found in the stomach, duodenum, and jejunum but case reports have described it being present in the esophagus, ileum, Meckel’s diverticulum, gallbladder, liver, and omentum as well [2]. On endoscopic ultrasound (EUS), pancreatic rest appears hypoechoic (or has mixed echogenicity) and usually arises from the second, third, or fourth layer (muscularis mucosa, submucosa, and muscularis propria, respectively) [3]. This case report was previously presented as an abstract at the American College of Gastroenterology, San Antonio, Texas, October 25-30, 2019, and the Pennsylvania Society of Gastroenterology, Pocono Manor, PA, October 11-13, 2019.

## Case presentation

**Figure 1:**
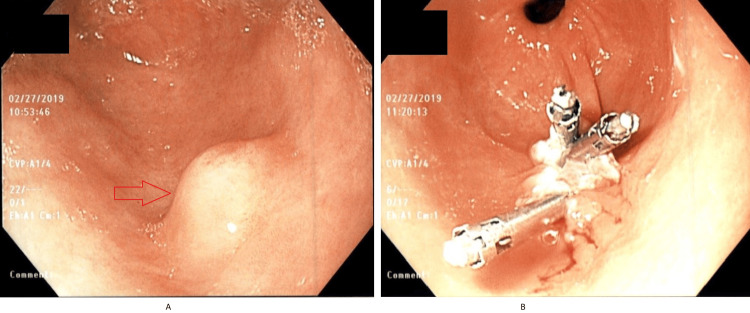
(a) EGD showing sub-epithelial lesion in the antrum (red arrow). (b) Follow-up EGD showing scarring in the area of EMR resection EGD, esophagogastroduodenoscopy; EMR, endoscopic mucosal resection

**Figure 2:**
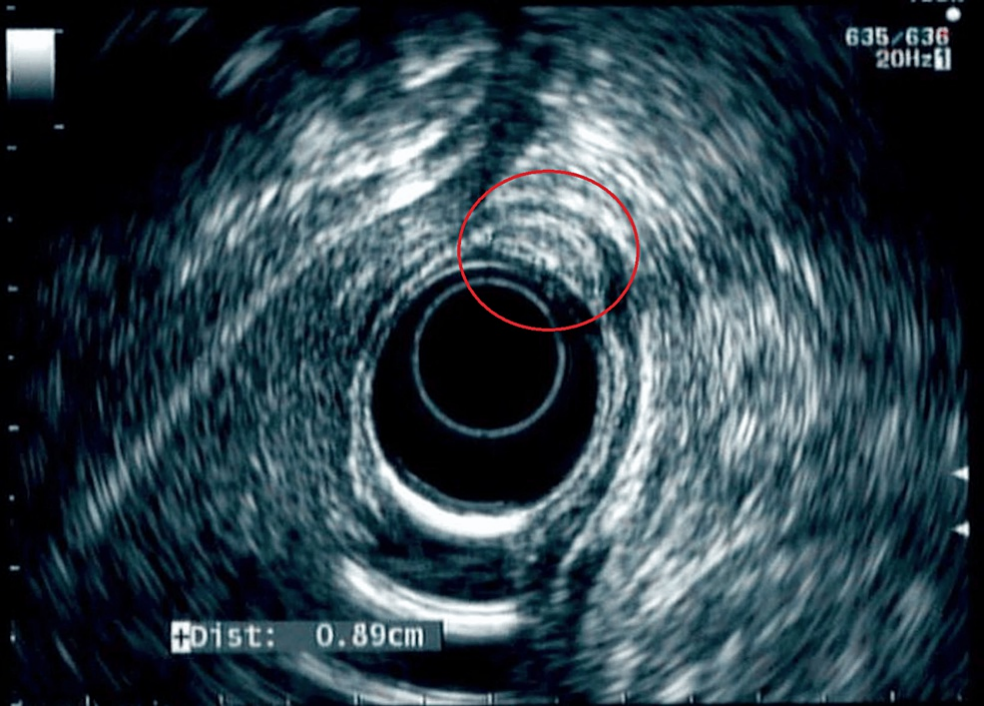
EUS showing nodule arising from muscularis mucosa, hypoechoic with heterogeneous areas (red circle) EUS, endoscopic ultrasound

**Figure 3:**
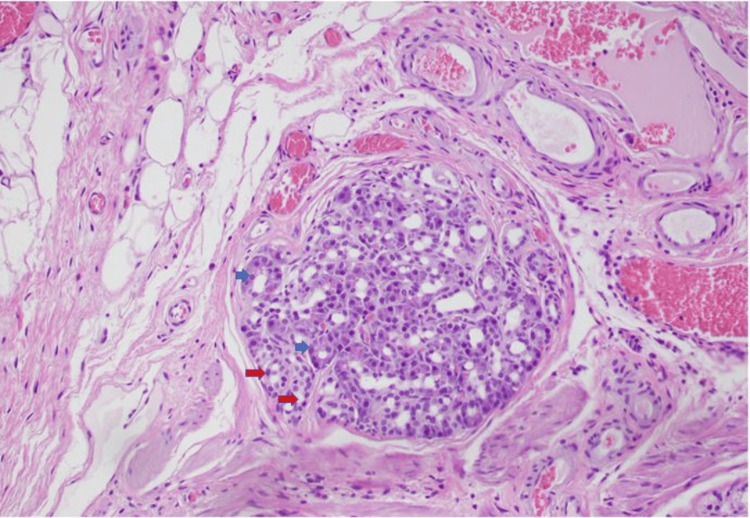
Pancreatic heterotopia within stomach wall (center) with surrounding muscularis layer. The pancreatic heterotopia shows the pancreatic acini (blue arrow) with associated duct (red arrow)

**Figure 4:**
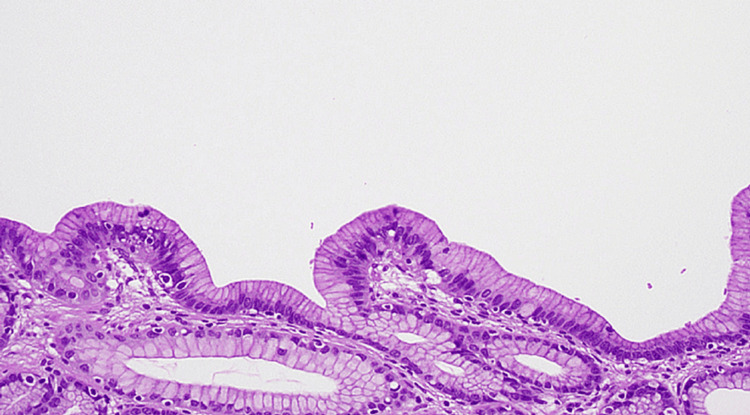
PanIN-1 with apical mucin PanIN-1, low-grade pancreatic intraepithelial neoplasia

An 82-year-old male with a past medical history of hypertension, gastroesophageal reflux disease, and coronary artery disease presented to the gastroenterology clinic with episodic abdominal pain that did not improve despite being on proton pump inhibitor (PPI) therapy. The pain was located in the epigastrium. He denied any symptoms of melena, weight loss, nausea/vomiting, or constipation/diarrhea. Physical examination revealed a soft, non-distended abdomen and mid-epigastric tenderness. Labs showed a normal complete blood count and comprehensive metabolic panel. His last esophagogastroduodenoscopy (EGD) was performed two years ago, and he was found to have an antral nodule. A repeat EGD was done and showed a sub-epithelial nodule in the gastric antrum measuring 7-8 mm that was unchanged from before (Figure 1a). The nodule was mobile. The patient was then scheduled for an EUS, which showed an intramural sub-epithelial lesion in the antrum arising from the muscularis mucosa (Figure 2). The lesion was hypoechoic but had some heterogeneous areas. It measured approximately 8 mm × 3 mm. No high-risk features were present. Given the heterogeneous appearance and patient’s symptoms, the area was resected using a Cook-Duette Multibanding EMR (endoscopic mucosal resection) kit. Post resection, three clips were applied. The EUS also incidentally revealed a cystic dilation of the pancreatic duct in the head of the pancreas measuring 5 mm. This was likely a main duct intra-papillary mucinous neoplasm. Results from the resection sample demonstrated the presence of pancreatic acini and ductal cells suggestive of pancreatic rest with focal low-grade PanIN (PanIN-1) and clear margins as well as reactive gastropathy with focal intestinal metaplasia (Figure 3 and Figure 4). He continued PPI therapy with the resolution of abdominal pain. A repeat EGD was done in one year that showed scarring in the area of the previous EMR and no evidence of recurrent nodules (Figure 1b).

## Discussion

**Figure 5:**
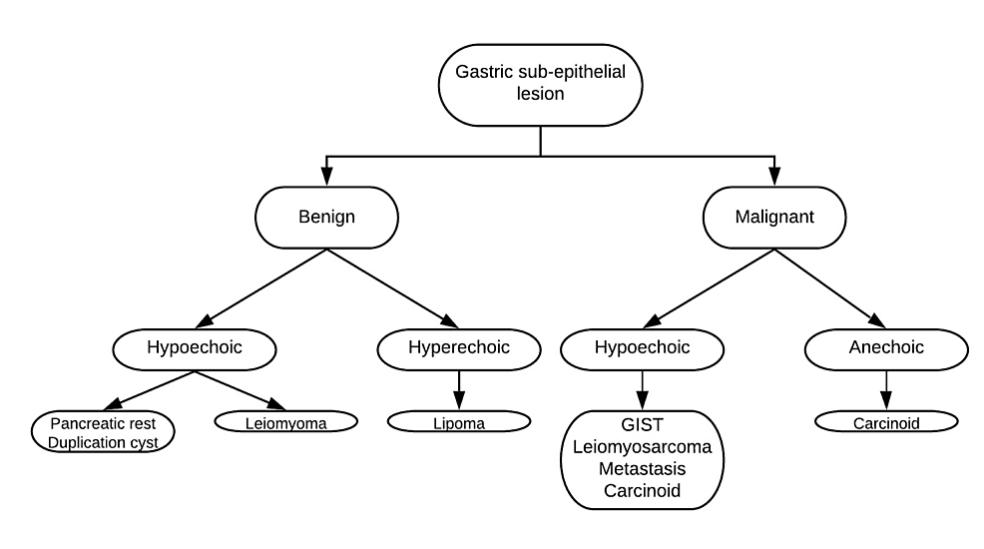
Classification of gastric sub-epithelial nodule This figure is a creation by the authors.

It is important to consider pancreatic rest as a differential for an intramural gastric sub-epithelial lesion. An EUS is an important modality to characterize gastric sub-epithelial lesions. It can determine the echogenicity, the layer of origin, and give us tissue for sampling. The differential diagnosis of the most common intramural gastric sub-epithelial lesions is listed in the flow chart below (Figure 5). These are categorized based on their findings on EUS.

Malignant features of an intramural lesion on EUS are a size greater than 3 cm, echogenic foci greater than 3 mm, cystic areas within the lesion, irregular borders, and the presence of lymph nodes [4]. Our patient’s lesion was predominantly hypoechoic but had some heterogeneous appearance and was arising from the muscularis mucosa. The presence of acini within the ectopic pancreatic tissue is known to give it a heterogeneous appearance. Even though our patient did not have any of these malignant features, given the heterogeneous appearance of the nodule and given his abdominal pain, a decision was made to resect the lesion using an EMR kit. EMR has shown to be superior compared to EUS with fine needle aspiration (FNA) in terms of diagnostic yield for gastric sub-epithelial lesions [5].

Pancreatic rest can be located virtually anywhere within the gastrointestinal tract, though they are more commonly found in the stomach. Specifically, they are most commonly located in the distal stomach along the greater curvature of the antrum. Although usually asymptomatic, the most commonly reported symptom is epigastric abdominal pain, occurring in as many as 75% of symptomatic cases. Reported complications of pancreatic rest in the stomach include acute or chronic pancreatitis, necrosis, pseudocyst, gastric outlet obstruction, and rarely carcinoma [6-8].

An important finding in our patient’s histology was the presence of PanIN-1. PanIN-1 is the presence of low-grade dysplasia that can progress to PanIN-3, high-grade dysplasia. Ultimately, there is a risk of development of pancreatic ductal adenocarcinoma (PDA) [9]. It is interesting to note that the genetic abnormalities found in PDA are also found in PanIN. These include mutations in K-ras, overexpression of cyclin D, accumulation of p53, and loss of expression of p16 [10]. This means that patients with pancreatic rest are at a higher risk of developing PDA in the ectopic tissue as well as in their native pancreas. Even though we resected our patient’s lesion, it is important to monitor him for the development of PDA in the future.

## Conclusions

The case report highlights the importance of considering pancreatic rest as a potential differential diagnosis for a gastric sub-epithelial lesion, particularly when patients present with abdominal pain. Resection of nodules with heterogeneous appearances should be considered, as demonstrated in this case, where seemingly benign nodules unexpectedly revealed pancreatic intraepithelial neoplasia. The case contributes to our understanding of pancreatic rest and the identification of PanIN-1 within ectopic pancreatic tissue emphasizes the need for long-term surveillance despite complete resection. 
